# Discovery of β-lactam-resistant variants in diverse pneumococcal populations

**DOI:** 10.1186/s13073-014-0072-8

**Published:** 2014-09-25

**Authors:** Regine Hakenbeck

**Affiliations:** Department of Microbiology, University of Kaiserslautern, Paul-Ehrlich Strasse, Gebäude 23, D-67663, Kaiserslautern, Germany; Alfried Krupp Wissenschaftskolleg Greifswald, D-17487 Greifswald, Germany

## Abstract

Understanding of antibiotic resistance in *Streptococcus pneumoniae* has been hindered by the low frequency of recombination events in bacteria and thus the presence of large linked haplotype blocks, which preclude identification of causative variants. A recent study combining a large number of genomes of resistant phenotypes has given an insight into the evolving resistance to β-lactams, providing the first large-scale identification of candidate variants underlying resistance.

*Streptococcus pneumoniae* has been causally attributed to many diseases, such as pneumonia, septicemia and meningitis. β-Lactam antibiotics such as penicillins and cephalosporins are the main form of treatment, but antibiotic resistance is a frequent problem for which the underlying mechanism remains elusive. A recent study by Julian Parkhill and colleagues [1] of more than 3,000 isolates of this pathogen examined which genes (and mutations therein) are responsible for the evolution of penicillin resistance. One might wonder why we need to obtain genome sequences from thousands of isolates for what might seem a simple question? In fact, the development of penicillin resistance in *S. pneumoniae* is far from being simple.

Unlike in many other bacterial pathogens, β-lactam resistance in pneumococci is not related to β-lactamase production; for unknown reasons β-lactamases have not been detected in *S. pneumoniae* so far. Instead, the resistance mechanism involves alterations in genes encoding target enzymes for β-lactam antibiotics, the penicillin-binding proteins (PBPs; reviewed in [2,3]). PBPs are crucial enzymes acting in the biosynthesis of peptidoglycan (PG), a major constituent of the bacterial cell wall. They are part of the machinery for cell elongation and division. The PG layer can be visualized as one huge macromolecule surrounding the entire cell; it maintains the shape of the cell and is required for its osmotic stability. PG is composed of glycan strands with alternating *N*-acetylglucosamine (GlcNAc) and *N*-acetylmuramic acid (MurNAc) residues, the latter containing short peptides. Elongation of the glycan strands and crosslinking of the peptides results in the network-like structure ubiquitously found in bacteria; these two reactions are carried out by different PBPs. Peptide crosslinking, a transpeptidation reaction, is the crucial penicillin-sensitive step carried out by the transpeptidase/penicillin-binding domain present in all PBPs. Because β-lactams are covalently bound through the active-site serine of the PBP, mutations in β-lactam-resistant strains reduce the protein's affinity for the antibiotic; that is, the mutated enzymes do not interact with the inhibitory drug even at higher concentrations, and PG biosynthesis can continue. Identification of the variants that cause resistance is thus important in defining the mechanism of resistance.

Research into causal variants is confounded by the following issues. First, at least three PBPs are required for high-level resistance in *S. pneumoniae*. Second, there are different mutational pathways that result in a low-affinity PBP. Third and most importantly, resistant clinical strains contain mosaic genes in which sections are replaced by highly altered sequences that differ by approximately 20% at the DNA level, the result of interspecies gene transfer followed by recombination events. This means that only a few of the mutations detected in the PBP genes are related to resistance [2]. As *S. pneumoniae* is a naturally transformable species, that is, it takes up DNA easily, so resistance determinants can be transferred easily within the population via intraspecies gene transfer, giving rise to new mosaic structures. Last but not least, non-PBP genes have been shown also to contribute to resistance.

In other words, within the pneumococcal population there is a highly variable gene pool of mosaic PBP and non-PBP genes, and each resistant clone may contain a distinct set of alleles responsible for the resistance phenotype. As a consequence, there is a wide range of resistance profiles, including preferential resistance for a subfamily of β-lactams such as cephalosporins. One example is the appearance of high-level cephalosporin-resistant strains in the USA, the consequence of one single mutation in the PBP2x gene [4].

Parkhill and colleagues [1] used a series of bioinformatic tools to identify single nucleotide polymorphisms (SNPs) and indels that are associated with β-lactam resistance. They studied two independent datasets derived from a collection of β-lactam-sensitive and -resistant *S. pneumoniae* samples from Thailand and the USA, two bacterial populations that have evolved independently and thus represent a very different spectrum of genotypes. In the era of genomics, it is extremely important to use appropriate tools to analyze big datasets, and this study is a perfect example of how to achieve a high-resolution genetic analysis from the genomes of more than 3,000 isolates. The large dataset allowed the narrowing down of large recombinant mosaic blocks to much smaller loci, enabling the identification of SNPs that may be causally associated. In total, 301 SNPs across 51 loci were found to be associated with non-susceptibility in both pneumococcal populations; these are preferential candidates for experimental testing. This included the confirmation of many previously identified loci and SNPs and 43 novel non-synonymous polymorphic changes, documenting the effectiveness of the method. Parkhill and colleagues [1] also demonstrated preferential association of the loci with resistance to specific subfamilies of β-lactams, penicillins or cephalosporins, suggesting that the underlying mechanisms may be different.

For basic research, the identification of new SNPs is important for our understanding of the mode of action of PBPs. But which SNPs should be tested in an experimental setting? Some SNPs might be associated with genes that are located close to the actual target as a result of a hitchhiking effect during gene transfer and recombination events rather than having a role in resistance itself. Parkhill and colleagues [1] identified a variety of genes besides those encoding PBPs. Among them, the genes *mraY* and *mraW* (encoding transferases in PG biosynthesis), *ftsL* (involved in cell division) and *clpL* (encoding a heat shock protein and chaperone) are located up- and downstream of *pbp2x*. Although *clpL* has been shown to be associated with penicillin susceptibility [5], further experimental testing is required to distinguish causative SNPs from hitchhiking SNPs by recombination, and the same is true for *recU*, located upstream of *pbp1a*.

Some SNPs might be compensatory mutations to reduce an adverse effect of primary resistance mutations in what are, after all, essential enzymes. Testing a mutation for its effect on resistance is also a difficult matter. Some of the mutations known to be important confer only a fairly moderate effect on susceptibility when introduced as a single mutation in the wild-type background. The effect of mutation on the activity of the enzymes themselves is not well known, and compensatory mutations may also occur in other genes. This includes components of the machinery responsible for PG synthesis and for cell division apparatus, pathways for which possible resistant variants were found in this study [1]. Most PBPs of *S. pneumoniae* are located at the division septum and are part of protein complexes required for division and cell elongation [6]. Because of this complexity, it will be necessary to analyze SNPs that are associated with both frequent and rare resistance pathways in individual lineages. Functional validation of the results will require extensive work, including the structural resolution of new variants of the cell wall synthesis and cell division machinery.

There are important issues of clinical importance. The SNPs identified here [1] are distributed not only in vaccine-targeted but also in non-vaccine-targeted lineages. The polysaccharide capsule is a major pathogenicity factor in pneumococci, and antibodies directed against it represent a major defense mechanism against this pathogen. Over 93 biochemically distinct capsule types are known [7], but only a few are common in humans. Protein-conjugated vaccination against the prevalent 7 and more recently 13 serotypes has been successfully implemented in many countries, leading to a reduction in infection, especially among children, with a major effect on invasive and non-invasive disease [8-10]. The fact that Chewapreecha *et al*. [1] now show that the SNPs are enriched not only in vaccine-targeted but also non-vaccine-targeted lineages is one potential explanation for why vaccination has not reduced β-lactam resistance within the population. Thus, some non-vaccine-targeted lineages containing a high frequency of resistant alleles can act as both a source and a sink of resistance alleles within the population.

Sequence-based detection of SNPs may be useful to predict penicillin susceptibility in clinical microbiology, providing rapid diagnostic testing required for optimal therapy. Moreover, this may pave the way to the development of antibiotics that target specific variants and could address the ever-evolving problem of the spread of antibiotic resistance.

## Abbreviations

PBP: Penicillin-binding protein
PG: Peptidoglycan
SNP: Single nucleotide polymorphism
